# Alterations of gut microbiota diversity, composition and metabonomics in testosterone-induced benign prostatic hyperplasia rats

**DOI:** 10.1186/s40779-022-00373-4

**Published:** 2022-03-28

**Authors:** Lu-Yao Li, Jie Han, Lan Wu, Cheng Fang, Wei-Guang Li, Jia-Min Gu, Tong Deng, Chang-Jiang Qin, Jia-Yan Nie, Xian-Tao Zeng

**Affiliations:** 1 Center for Evidence-Based and Translational Medicine, Zhongnan Hospital of Wuhan University, Wuhan, 430071 China; 2 Department of Gastrointestinal Surgery, Huaihe Hospital of Henan University, Kaifeng, 475000 Henan China; 3 Department of Gastroenterology, Zhongnan Hospital of Wuhan University, Wuhan, 430071 China; 4 Department of Stomatology, Zhongnan Hospital of Wuhan University, Wuhan, 430071 China; 5 Department of Urology, Zhongnan Hospital of Wuhan University, Wuhan, 430071 China

**Keywords:** Benign prostatic hyperplasia, Gut microbiota, Intestinal metabolites, Microbial diversity

## Abstract

**Background:**

Studies had shown many diseases affect the stability of human microbiota, but how this relates to benign prostatic hyperplasia (BPH) has not been well understood. Hence, this study aimed to investigate the regulation of BPH on gut microbiota composition and metabonomics.

**Methods:**

We analyzed gut samples from rats with BPH and healthy control rats, the gut microbiota composition and metabonomics were detected by 16S rDNA sequencing and liquid chromatography tandem mass spectrometry (LC–MS/MS).

**Results:**

High-throughput sequencing results showed that gut microbiota beta-diversity increased (*P* < 0.01) in the BPH group vs. control group. *Muribaculaceae* (*P* < 0.01), *Turicibacteraceae* (*P* < 0.05), *Turicibacter* (*P* < 0.01) and *Coprococcus* (*P* < 0.01) were significantly decreased in the BPH group, whereas that of *Mollicutes* (*P* < 0.05) and *Prevotella* (*P* < 0.05) were significantly increased compared with the control group. Despite profound interindividual variability, the levels of several predominant genera were different. In addition, there were no statistically significant differences in several bacteria. BPH group vs. control group: *Firmicutes* (52.30% vs. 57.29%, *P* > 0.05), *Bacteroidetes* (46.54% vs. 41.64%, *P* > 0.05), *Clostridia* (50.89% vs. 54.66%, *P* > 0.05), *Ruminococcaceae* (25.67% vs. 20.56%, *P* > 0.05). LC–MS/MS of intestinal contents revealed that differential metabolites were mainly involved in cellular processes, environmental information processing, metabolism and organismal systems. The most important pathways were global and overview maps, lipid metabolism, amino acid metabolism, digestive system and endocrine system. Through enrichment analysis, we found that the differential metabolites were significantly enriched in metabolic pathways, steroid hormone biosynthesis, ovarian steroidogenesis, biosynthesis of unsaturated fatty acids and bile secretion. Pearson correlation analysis (*R* = 0.94) showed that there was a strong correlation between *Prevotellaceae*, *Corynebacteriaceae*, *Turicibacteraceae*, *Bifidobacteriaceae* and differential metabolites.

**Conclusion:**

Our findings suggested an association between the gut microbiota and BPH, but the causal relationship between the two groups is unclear. Thus, further studies are warranted to elucidate the potential mechanisms and causal relationships between BPH and gut microbiota.

**Supplementary Information:**

The online version contains supplementary material available at 10.1186/s40779-022-00373-4.

## Background

Gut microbiota co-evolve with humans and make significant contributions to human biology and development [1]. Over the past decade, there has been a growing understanding of the important role of the human gut microbiome in health and disease. The human gut microbiome is made up of about 40 trillion microbes, including up to 1000 different microbial species [2, 3]. In general, the continuous competition and interaction among microorganisms may lead to a gradual change in the structure of microbial communities from simple to complex, and eventually to a dynamically balanced ecosystem [4]. Many animal and human studies have shown that intestinal microbiota plays an important role in growth and development, metabolism, immune regulation, and promoting host health. Due to the close symbiotic relationship between intestinal microbiota and host, dysregulation of gut microbiome has been linked to diseases in many systems or the suggestion that the microbiome was influenced by these diseases, such as the gut microbial markers of type 2 diabetes may be useful for classifying type 2 diabetes, and the bacterial markers of type 2 diabetes may affect the intestinal flora [5, 6]; Crohn's disease reduces the diversity of gut microbes [7, 8]. Colorectal cancer was associated with decreased bacterial diversity in feces [9]. Therefore, looking for factors that affect the homeostasis of the intestinal flora is of great significance for us to further understand the intestinal flora and prevent and treat intestinal-related diseases.

Benign prostatic hyperplasia (BPH) is one of the most common diseases of older men, affecting about 42% by age fifty and about 80% by age eighty [10], this proportion increased to 90% by age 85 [10–12]. Men with BPH usually exhibit a range of lower urinary tract symptoms (LUTS), including frequent and urgent urination, nocturia, urine hesitance, and reduced urination [10, 13], which can lead to urinary dysfunction and have a negative impact on quality of life [14]. BPH has been considered as a complex inflammatory condition which is influenced by the oral and gut microbiota [15–18]. However, it is not clear whether BPH status can influence the intestinal flora. Hence, we use animal models to study the effects of BPH on gut microbiota composition and metabonomics, from the level of 16S rDNA sequencing and liquid chromatography tandem mass spectrometry (LC–MS/MS).

## Methods

### Source of animals and study design

The male SPF grade SD rats (7-week-old, *n* = 10) were provided by the Beijing Vital River Laboratory Animal Technology Co. Ltd. Rats were kept in constant environmental conditions of humidity (55 ± 10)% and temperature (22 ± 2)°C on a 12-h light/dark cycle and had unrestricted access to water and food. All animals were randomly distributed to the cages by an animal facilities technician, and before any procedure, the cages were selected randomly and randomized to each group by a person not involved in the study. All the in vivo experiments as well as the investigators responsible for data collection and analysis were blinded. The animals were cared for in accordance with the “Guide for the care and use of laboratory animals” in China. This study was approved by the Animal Ethics Committee of Wuhan University (IACUC 2018119), and all efforts were made to minimize animal suffering.

### Experimental model of BPH

SD rats were adaptively fed for one week in the animal house, then were divided into two groups according to a randomized block design: control group (*n* = 5), and BPH group (*n* = 5). The experiments were carried out according to established methods. The rats in BPH group were anesthetized and castrated. A week later, testosterone (5 mg/kg) was administered to the rats of the BPH group once a day for 4 weeks [19, 20]. The rats in control group received no intervention. The weight of all rats was measured and feces were collected from each rat after the intervention. Feces were collected in sterile cryopreservation tubes, and then labeled and stored in a − 80 °C refrigerator. The rats were anesthetized with an intraperitoneal injection of pentobarbital sodium and sacrificed, then prostate tissue samples from each rat were separated and the wet weight of the prostate tissue was measured. Prostate tissue was fixed with 4% formaldehyde, and tissue sections were subsequently made.

### HE and masson staining

For histological evaluation, all the rat prostate paraffin blocks were cut into Sects. 3 - 5 μm thick. For HE staining, the sections were stained with haematoxylin for 10 min and then stained with eosin for 15 min. For Masson staining, the sections were stained with haematoxylin for 10 min, Ponceau for 10 min, phosphomolybdic acid for 2 min, and aniline blue for 2 min.

### DNA extraction, 16S rDNA amplification and sequencing analysis

The microbial community DNA was extracted using MagPure Stool DNA KF kit B (Magen, China) following the manufacturer's instructions. DNA was quantified with a Qubit Fluorometer by using Qubit dsDNA BR Assay kit (Invitrogen, USA) and the quality was checked by running aliquot on 1% agarose gel.

Variable regions V3-V4 of bacterial 16S rRNA gene was amplified with degenerate PCR primers, 341F (5’-ACTCCTACGGGAGGCAGCAG-3’) and 806R (5’-GGACTACHVGGGTWTCTAAT-3’). PCR cycling conditions were as follows: 94 °C for 3 min, 30 cycles of 94 °C for 30 s, 56 °C for 45 s, 72 °C for 45 s and final extension for 10 min at 72 °C. The PCR products were purified with AmpureXP beads and eluted in Elution buffer. Libraries were qualified by the Agilent 2100 bioanalyzer (Agilent, USA). The validated libraries were used for sequencing on Illumina MiSeq platform (BGI, Shenzhen, China) following the standard pipelines of Illumina.

Clean data was obtained by processing the original sequencing data. The specific steps are as follows: (1) Reads that can be matched with primers are intercepted with Cutadapt V2.6 software to obtain fragments of the target region. (2) The method of removing low quality by window was adopted: 30 bp was set as the window length. If the window average quality value was lower than 20, the read end sequence was removed from the window and the reads whose final read length was lower than 75% of the original read length were removed. (3) Remove reads containing N. (4) The low-complexity reads were removed to obtain the final clean data. After removing adaptors and low-quality and ambiguous bases, the paired-end reads were added to tags by the Fast Length Adjustment of Short reads program (FLASH, v1.2.11) to get the tags. The tags were clustered into operational taxonomic units (OTUs) with a cutoff value of 97% [21] using UPARSE software (v7.0.1090) and chimera sequences were compared with the Gold database using UCHIME (v4.2.40) for detection. Then, OTU representative sequences were taxonomically classified using Ribosomal Database Project (RDP) Classifier v2.2 with a minimum confidence threshold of 0.6, and trained on the Greengenes database [22] v201305 by QIIME v1.8.0 [23]. USEARCH global was used to compare all tags back to OTU to get the OTU abundance statistics table of each sample.

Sequencing analysis includes alpha diversity analysis, beta diversity analysis and different taxa analysis. Alpha diversity analysis was the analysis of taxa diversity within a single sample. Chao index and Shannon index were used to evaluate the microbiota richness and evenness of a single sample. Beta diversity analysis was conducted based on weighted and unweighted UniFrac distance matrix [24]. Beta diversity analysis was carried out to compare the differences in microbial composition between groups. Linear discriminant analysis effect size (LEfSe) difference analysis was used to identify taxa with significant differences by LEfSe [25]. Alpha and beta diversity were estimated by MOTHUR (v1.31. 2) and QIIME (v1.8.0) at the OTU level, respectively. Sample cluster was conducted by QIIME (v1.8.0) based on unweighted pair group method with arithmetic mean (UPGMA). Kyoto encyclopedia of genes and genomes (KEGG) and clusters of orthologous genes (COG) functions were predicted using the PICRUSt software. Barplot and heatmap of different classification levels were plotted with R package v3.4.1 and R package “gplots”, respectively.

### LC–MS/MS analysis

We used a Waters 2D ultra performance liquid chromatography (UPLC) (Waters, USA) tandem Q Exactive HF high resolution mass spectrometer (Thermo Fisher Scientific, USA) for separation and detection of metabolites. A 50 μl sample was extracted from each gut specimen and mixed to make quality control (QC) samples [26]. To provide more reliable experimental results during instrument testing, the samples are randomly ordered to reduce system errors. A QC sample is interspersed for every 10 samples [27].

The samples were analyzed on a Waters 2D UPLC, coupled to a Q-Exactive mass spectrometer (Thermo Fisher Scientific, Waltham, MA, USA) with a heated electrospray ionization (HESI) source and controlled by the Xcalibur 2.3 software program (Thermo Fisher Scientific, Waltham, MA, USA). Chromatographic separation was performed on a Waters ACQUITY UPLC BEH C18 column (1.7 μm, 2.1 mm × 100 mm, Waters, USA), and the column temperature was maintained at 45 °C. The mobile phase consisted of 0.1% formic acid (A) and acetonitrile (B) in the positive mode, and in the negative mode, the mobile phase consisted of 10 mmol/L ammonium formate (A) and acetonitrile (B). The gradient conditions were as follows: 0–1 min, 2% B; 1–9 min, 2–98% B; 9–12 min, 98% B; 12.0–12.1 min, 98% B to 2% B; and 12.1–15.0 min, 2% B. The flow rate was 0.35 ml/min and the injection volume was 5 μl.

The mass spectrometric settings for positive/negative ionization modes were as follows: spray voltage, the positive ion mode is 3.8 kV, and the negative ion mode is 3.2 kV; sheath gas flow rate, 40 arbitrary units (arb); aux gas flow rate, 10 arb; aux gas heater temperature, 350 °C; capillary temperature, 320 °C. The full scan range was 70–1050 m/z with a resolution of 120,000, and the automatic gain control (AGC) target for MS acquisitions was set to 3e6 with a maximum ion injection time of 100 ms. Top 3 precursors were selected for subsequent MSMS fragmentation with a maximum ion injection time of 50 ms and resolution of 30,000, the AGC was 1e5. The stepped normalized collision energy was set to 20, 40 and 60 eV.

The mass spectrometry raw data (raw file) collected by LC–MS/MS was imported into Compound Discoverer 3.1 (Thermo Fisher Scientific, USA) for data processing, this mainly included peak extraction, peak alignment, and compound identification. Data pre-processing, statistical analysis, metabolite classification annotations and functional annotations were performed using the self-developed metabolomics R package metaX and the metabolome bioinformatic analysis pipeline. The multivariate raw data was dimensionally reduced by principal component analysis (PCA) to analyze the groupings, trends (intra-group and inter-group similarities and differences) and outliers of the observed variables in the data set (whether there is an abnormal sample). Using Partial Least Squares Method-Discriminant Analysis (PLS-DA), the Variable Importance in Projection (VIP), the variability analysis, the fold change and the Student's *t* test to screen for differential metabolites. During the analysis, the data were log_2_ transformed and z-score normalized (zero-mean normalization). The clustering algorithm used hierarchical cluster, and the distance calculation was performed in Euclidean distance. Metabolic pathway enrichment analysis of differential metabolites was performed based on the KEGG database. Metabolic pathways with *P* < 0.05 were significantly enriched by differential metabolites.

### 16S and metabolome correlation analysis

The metabolites obtained under the positive and negative ion collection mode were combined. When more than 200 metabolites were identified in the metabolome, Weighted Gene Co-expression Network Analysis (WGCNA) was used to reduce the dimensionality of the metabolome data. Rank correlation analysis, namely Spearman correlation analysis, was used to evaluate the correlation between two variables (metabolite abundance and microbial abundance). Spearman correlation analysis was performed on the metabolite co-expression cluster, the metabolic pathway to which the metabolites belong, and the differential metabolites with microorganisms at different levels, respectively, to reveal the correlation between the two omics in different dimensions and resolutions. Corr. test in R software was used to perform this analysis. Canonical correlation analysis (CCA) was used to calculate the overall correlation between metabolites and microorganisms, and to identify highly correlated biomarkers with classification effect. This project uses mixOmics in R software to implement canonical correlation analysis.

### Statistical analysis

The data were expressed as the means ± standard error of mean (SEM). Statistical analysis was performed using SPSS 17.0 software (SPSS, Inc., Chicago, IL, USA). The differences between groups were analyzed with an independent-sample *t* test, Wilcoxon rank-sum test or Mann–Whitney *U* test; Correlations were identified by Spearman’s rank correlation coefficient (significance thresholds were *P* < 0.05) using the corrplot package in R (v3.4.1). GraphPad Prism v8.0 (GraphPad Software Inc., San Diego, CA, USA) and R are used for various analyses and chart preparation. *P* < 0.05 was considered statistically significant.

## Results

### Establishment of BPH model

**Fig. 1:**
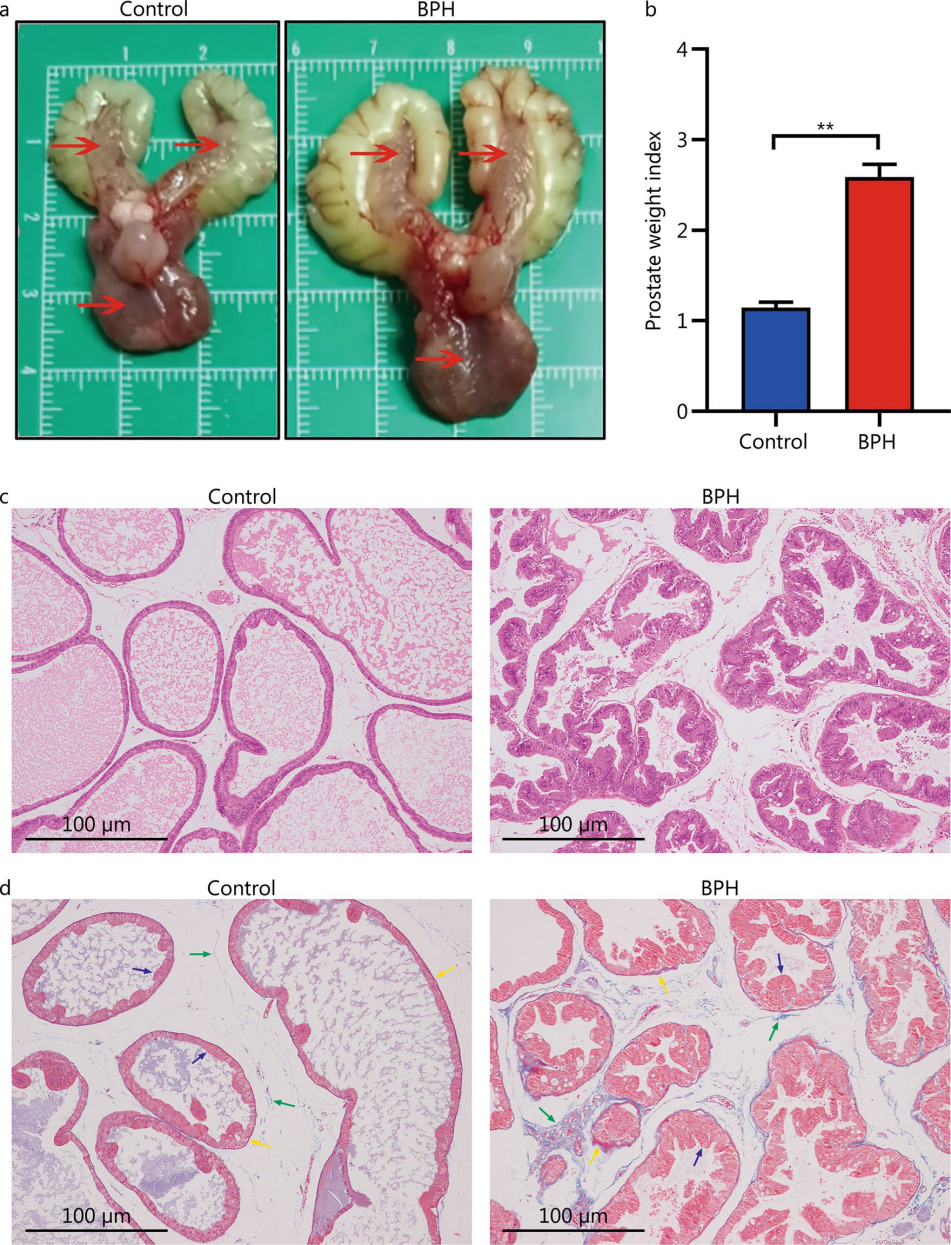
Pathological model of BPH in rats. **a** Anatomical map of rat prostate specimen (red arrows show the prostate tissue of a rat). **b** Prostate weight indexes were calculated dividing prostate weight (mg) by body weight (g). Results are expressed as mean ± SEM, **P < 0.01. HE staining (**c**) and Masson staining (**d**) of histopathological images of the prostate glands in each group [Masson staining: prostatic epithelial cells (blue arrow), smooth muscle cells (yellow arrow), and collagen fibers (green arrow)]. BPH: benign prostatic hyperplasia

In the BPH group, the appearance of the prostate tissue in rats was significantly larger than that in control group (Fig. 1a). The value of the prostate weight [(1009.40 ± 26.10) mg] was higher than that in the control group [(481.50 ± 26.50) mg] (*P* < 0.01). Prostate weight index, which could more accurately reflect the prostatic hyperplasia, was significantly larger than that in the control group (*P* < 0.01, Fig. 1b). The histological morphology of the prostate tissue in the BPH group was abnormal: the prostate connective tissue was increased in shape and size, the epithelial cell layer and lumen space in the BPH group were increased compared to those of the control group (Fig. 1c). Interacinar fibrosis was observed in the prostate gland in BPH group, and the amount of fibrous connective tissue was increased compared with that in control group (Fig. 1d). The above results indicated that the BPH model had been achieved.

### BPH is associated with alteration of gut microbiota diversity

#### Sequencing depth and quality are up to standard

In the microbiome analysis, a total of 707,592 and 706,930 original sequences were acquired from the BPH and control groups, respectively (Additional file 1: Table S1). After eliminating the unqualified data, a total number of 682,160 clean reads were obtained from the control group, and 684,936 clean reads were obtained from the BPH group, with an average of 96.65% (ranging from 96.21% to 97.04%) effective per sample (Additional file 1: Table S1). There was no statistical difference in the number of reads in sequencing data between the two groups (*P* > 0.05) (Additional file 2: Fig. S1a). Then, a total of 4412 OTUs (control = 2156, BPH = 2256) were identified based on 97% nucleotide sequence similarity from reads respectively (Additional file 1: Table S1). The OTUs were also not significantly different between the groups (*P* > 0.05) (Additional file 2: Fig. S1b), indicating that they presented similar microbiome richness.

To prove that all samples were sequenced to achieve excellent sequence depth and richness. The rarefaction and species accumulation curves for all the samples tended to be stable (Additional file 2: Fig. S1c). The curves of observed species could reflect the evenness, richness and diversity of each sample sequence. The number of qualified sequences reached 50,000, suggesting that sequencing’s depth and quantity met the demands for sequencing and analysis and covered most of the diversity. Furthermore, the OTU rank abundance curve was wide and falling gently, showing excellent abundance and evenness (Additional file 2: Fig. S1d).

#### BPH may affect inter-group diversity (beta-diversity) but not intra-group individual diversity (alpha-diversity)

**Fig. 2:**
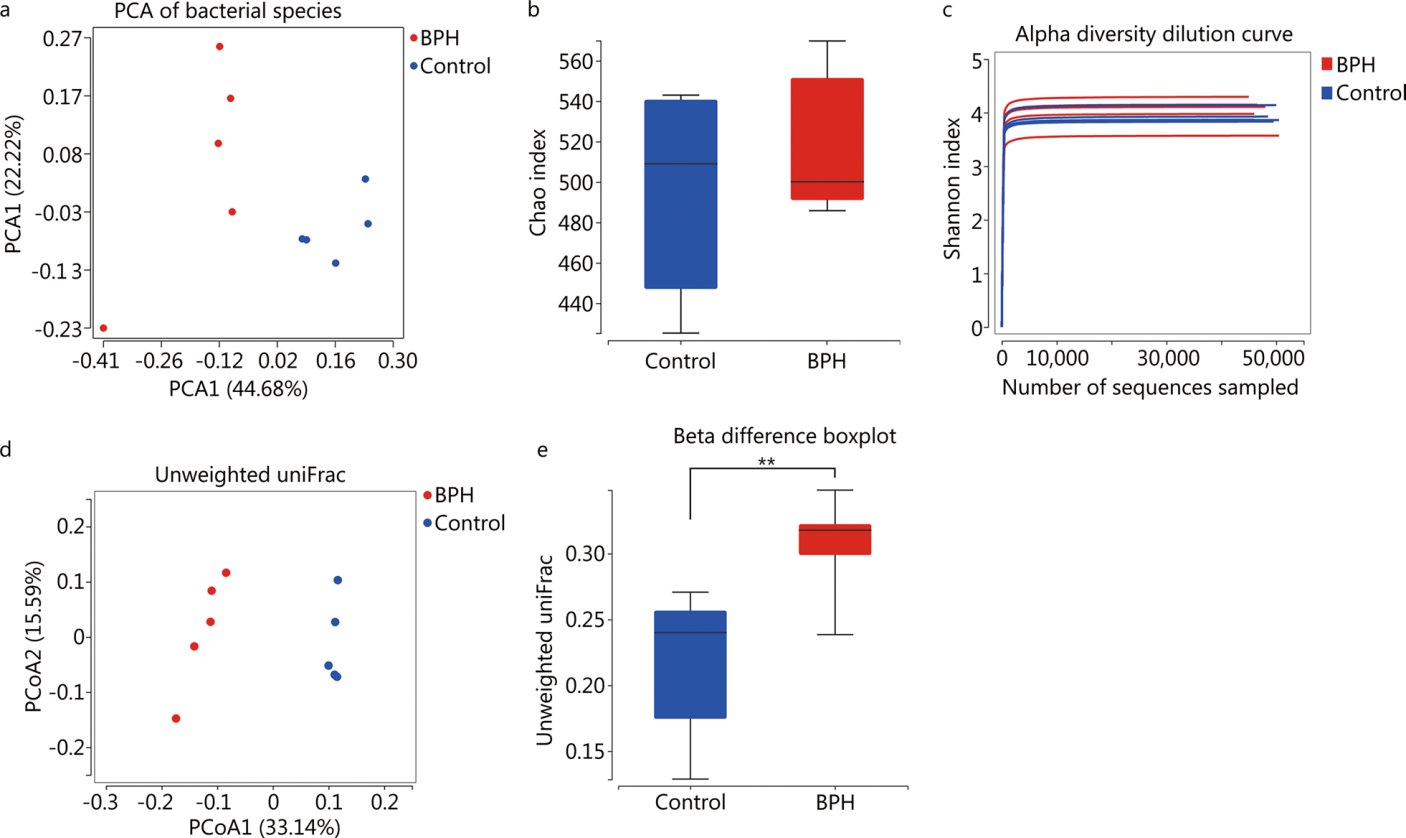
BPH affect inter-group diversity but not intra-group individual diversity. **a** Principal components analysis (PCA) of fecal microbiota from control and BPH group. **b**, **c** Box plots (Chao index) and dilution curve (Shannon index) show alpha diversity in gut microbiota of control and BPH rats, which reflect the richness and species diversity of each sample. Differences were assessed by non-parametric Wilcox test. **d** Principal coordinate analysis (PCoA) analysis of fecal microbiota from control and BPH based on unweighted UniFrac distance. The distinct clustering of samples was observed. The percentage of variation explained by PCoA1 and PCoA2 are noted in the axes. Groups are distinguished by colors. Each colored symbol corresponds to an individual sample. **e** Box plots showing beta diversity in fecal microbiota of control and BPH group (**P < 0.01). BPH: benign prostatic hyperplasia

The PCA analysis showed that gut samples in the control and BPH groups were separated into clusters, indicating that gut microbiota in the two groups was different (Fig. 2a). Alpha-diversity reflected the species richness and internal diversity of each individual sample, which was determined by several indexes. Chao index and Simpson index were calculated. We used the Chao index to estimate the gut microbial richness, which simply referred to the number of species in the community. We found that there was no statistical difference in Chao index between the two groups (*P* = 0.55, Fig. 2b), but it showed an upward trend in BPH, which may indicate that the richness of gut microbiota in BPH group was slightly higher than that in control group. Shannon index of the gut microbiota showed a downward trend in the BPH group compared to control group, but the differences were not significant (*P* = 0.31, Fig. 2c).

Unlike alpha-diversity, beta-diversity shows the variations of microbial communities between samples or groups. Principal coordinate analysis (PCoA) plots based on the unweighted UniFrac distance shows that gut microbial communities of the two groups are clearly separated into different clusters (Fig. 2d), and the beta difference boxplot shows significant differences between two groups (*P* < 0.01, Fig. 2e). Those indicate that BPH has significant effect on the diversity of intestinal flora in rats.

#### Gut microbiota composition is altered between the two groups

**Fig. 3:**
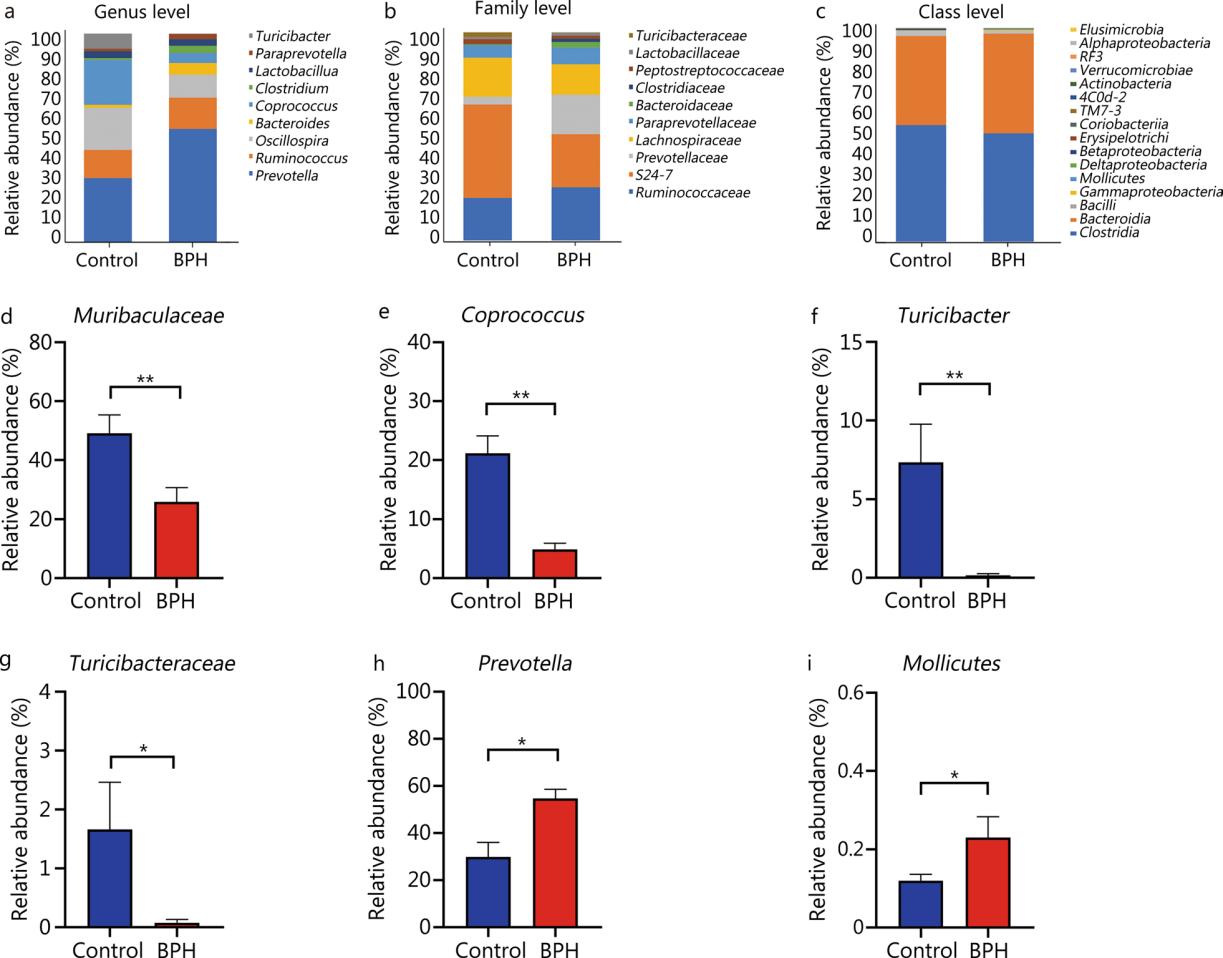
BPH altered the gut microbiota composition. Composition of fecal microbiome showing different communities at the genus (**a**), family (**b**) and class (**c**) level, different colors correspond to different species, and the color block length indicates the relative abundance of the species represented by the color. Relative abundance of (**d**) *Muribaculaceae* (family), **e** *Coprococcus* (genus), **f** *Turicibacter* (genus), **g** *Turicibacteraceae* (family), **h** *Prevotella* (genus), **i** *Mollicutes* (class). In (**d**–**i**) Mann–Whitney *U* test was used to determine statistically significant differences between the groups. Results are expressed as mean ± SEM, *P < 0.05; **P < 0.01. BPH: benign prostatic hyperplasia, OTUs: operational taxonomic units

To analyze the reasons for the differences in gut microbiota diversity and explain more detailed changes in the gut microbial structure between the two groups, we used bar charts to show the composition of the dominant microflora in the two groups and observe the significant changes of the microflora at different levels. Analysis of the gut microbiome also showed different compositions and abundance at the genus, family and class levels respectively (Fig. 3a–c). We found that compared with the control group, S24-7/*Muribaculaceae*[28], *Coprococcus*, *Turicibacter*, and *Turicibacteraceae* were significantly decreased in the BPH group (*P* < 0.05 or *P* < 0.01, Fig. 3d–i), and *Prevotella* and *Mollicutes* were significantly increased (*P* < 0.05, Fig. 3h, i). The predominant bacteria were *Firmicutes* and *Bacteroidetes* at the phylum level (Additional file 2: Fig. S1e), although there were no statistically significant differences for *Firmicutes* and *Bacteroidetes* (*P* > 0.05), *Firmicutes* (52.30% vs. 57.29%) showed a decreased trend and *Bacteroidetes* (46.54% vs. 41.64%) showed an increased trend compared with the control group. At the class level, compared with the control group, *Mollicutes* were significantly increased (*P* < 0.05, Fig. 3i), while *Clostridia* (50.89% vs. 54.66%, *P* > 0.05, Fig. 3c) showed a decreased trend in BPH group. At the family level, *Muribaculaceae* and *Turicibacteraceae* were significantly decreased in BPH group (*P* < 0.01, *P* < 0.05, Fig. 3d, g), while *Ruminococcaceae* (25.67% vs. 20.56%, *P* > 0.05, Fig. 3b) showed an increased trend compared with the control group. At the genus level, *Coprococcus* and *Turicibacter* were significantly decreased in BPH group (*P* < 0.01, Fig. 3e, f), but *Prevotella* were significantly increased compared with the control group (*P* < 0.05, Fig. 3h).

**Fig. 4:**
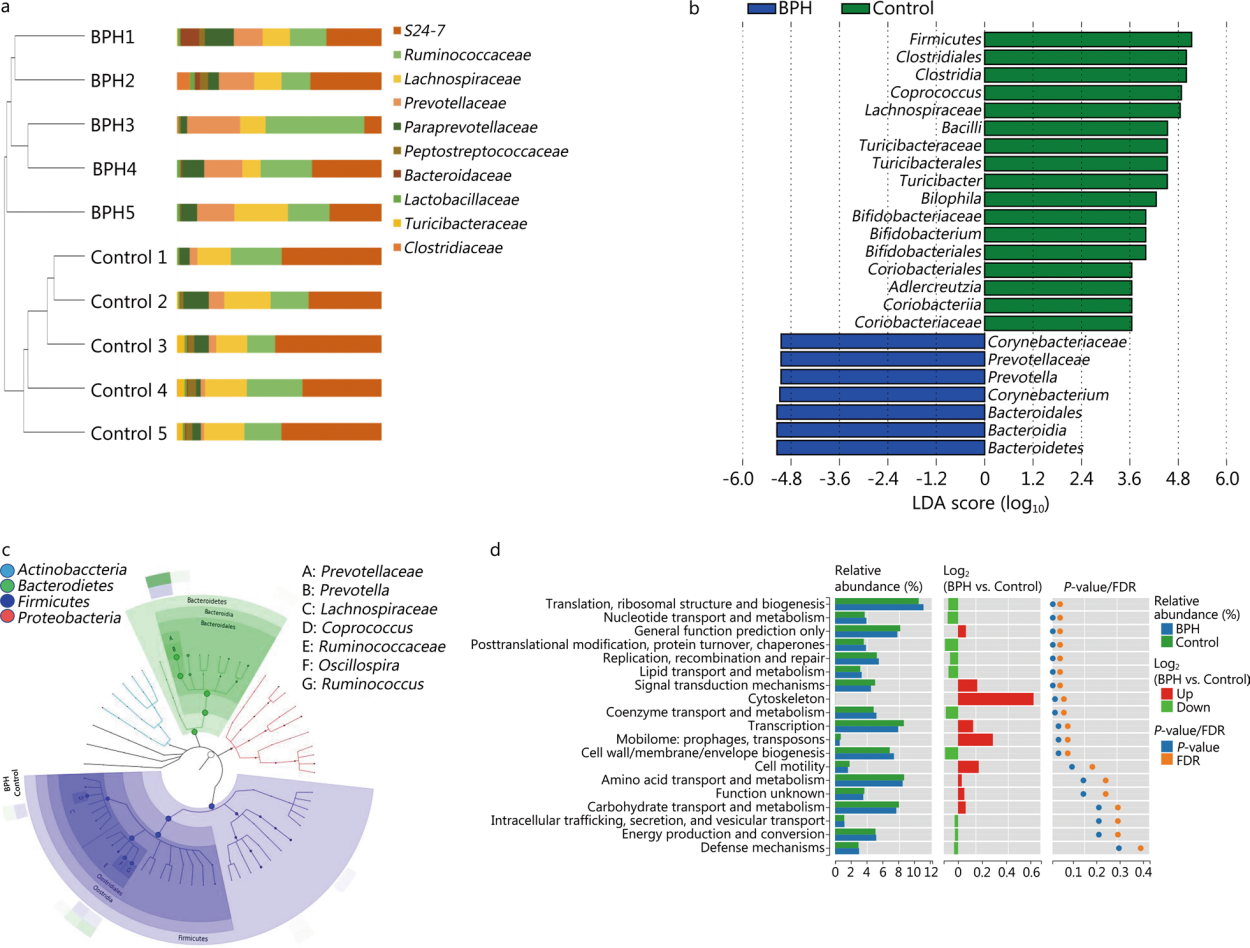
BPH regulates functional metabolism of the host by altering gut bacterial composition. **a** Unweighted pair group method with arithmetic mean (UPGMA cluster tree and abundance combination chart at family level: On the left is the result of the UPGMA clustering tree, and on the right is the histogram of species abundance). For samples under the same branch, the shorter the branch length between the samples, the more similar the two samples, and the higher the species composition similarity; the farther the distance, the greater the difference in species composition. **b** LDA diagram: Different colors represent groups of microorganisms that have significant effects in different groups. It mainly shows the significantly different species with LDA score > 2. The color of the histogram represents the respective group, and the length represents the LDA score, that is, the impact of significantly different species between different groups. **c** Species composition graph show species composition at each taxonomic level. Different colors indicate different groups. Nodes of different colors indicate the microbes that play an important role in the group represented by the color. From the inside to the outside, each circle is the species at the level of phylum, class, order, family, and genus. **d** Path difference diagram: The left side shows the relative abundance histogram of each group; the middle is the log_2_ value of the relative abundance mean ratio of the same passage in the two groups; the right side is the *P*-value and FDR value obtained by the Wilcox test. If *P* < 0.05 and FDR < 0.05, the pathway is significantly different between the two groups. BPH: benign prostatic hyperplasia, LDA: linear discriminant analysis, FDR: false discovery rate

To visually display the species composition and differences of the samples, according to the differences in results of each sample, the UPGMA clustering method was adopted to carry out clustering analysis on the samples at family class level (Fig. 4a). LEfSe analysis [α = 0.05, linear discriminant analysis (LDA) score > 2.0] coupled with a Species Composition Graph was performed to further compare the differences in intestinal microflora between the two groups (Fig. 4b, c). BPH group displayed a significant increase in the abundance of *Corynebacteriaceae*, *Prevotellaceae*, *Bacteroidales*, *Bacteroidia*, *Bacteroidetes*, *Prevotella*, *Corynebacterium* as well as a distinct decrease in the abundance of *Firmicutes*, *Clostridiales*, *Clostridia*, *Bacilli*, *Turicibacteraceae*, *Turicibacterales*, *Bifidobacteriaceae*, *Bifidobacteriales*, *Coriobacteriales*, *Coriobacteriia*, *Coriobacteriaceae*, *Lachnospiraceae*, *Turicibacter*, *Bilophila*, *Bifidobacterium*, *Adlercreutzia*, and *Coprococcus*.

We mapped the 16S sequencing results with the functional genes in the database to obtain the COG function prediction results. Wilcox test was used to look for the differences in functions between the groups after predicting the functions of all the samples, and the graph is presented (Fig. 4d). We found that the following functions were higher in the BPH group than that in the control group: translation, ribosomal structure and biogenesis, nucleotide transport and metabolism, posttranslational modification, protein turnover, chaperones, replication, recombination and repair, lipid transport and metabolism (*P* < 0.05). However, coenzyme transport and metabolism and signal transduction mechanisms were lower than those of the control group (*P* < 0.05).

### BPH may alter the metabolism of gut microbiota

#### Intestinal metabolites are different between the two groups

The intestinal gut samples were analyzed using the untargeted metabolomics approach through LC–MS/MS technology. Three visualization methods were used to control the quality of the data. The content includes base peak chromatogram (BPC), PCA and coefficient of variation (CV). We used a high-resolution mass spectrometer to collect data from both positive and negative ions to improve metabolite coverage. In the positive ion mode, the BPC of all QC samples were overlapped and the spectra were well overlapped (Additional file 2: Fig. S2a). PCA score plot showed all QC samples clustered near the origin in the PCA plot indicating good reproducibility (Additional file 2: Fig. S2b). The CV plot for QC samples showed that the proportion of positive ions (CV < 30%) reached 60% (Additional file 2: Fig. S2c). The QC sample tests results of negative ions were like those of positive ions (Additional file 2: Fig. S2d-f).

**Fig. 5:**
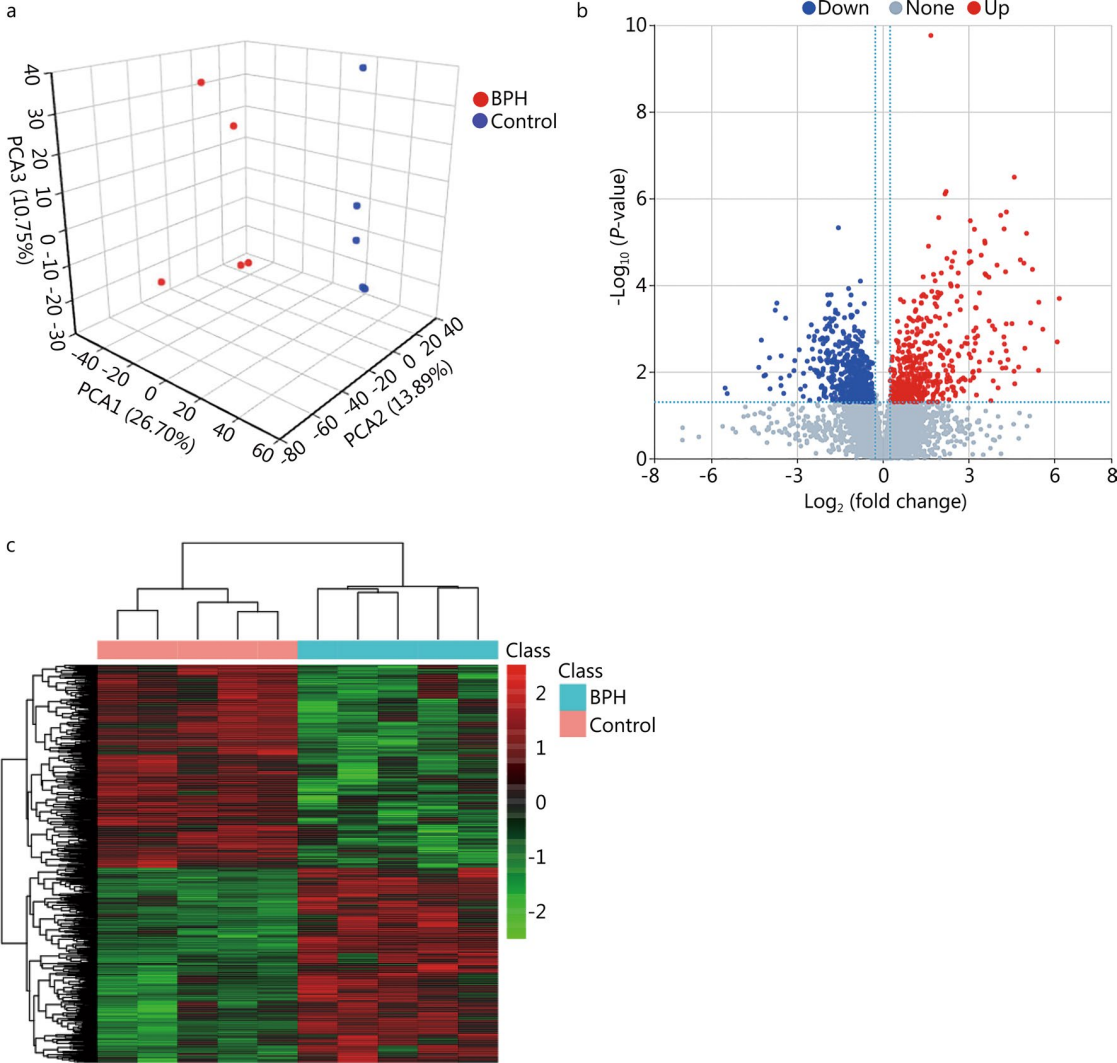
BPH may cause changes in intestinal metabolites. **a** PCA of positive ion compounds. Each point represents a sample, and different groups are marked with different colors. **b** Volcano map of different metabolites (positive ion compound): Blue represents the down-regulated differential metabolite, red represent the up-regulated differential metabolite, and metabolites without difference are labeled gray. **c** Cluster analysis of differential metabolites (positive ion compound): Each row in the figure represents a differential metabolite, and each column represents a sample. The color represents the expression level, and the green to red corresponds to the expression level from low to high. BPH: benign prostatic hyperplasia, PCA: principal component analysis

We performed the analysis and screening of differential ions between the BPH and control groups. PLS-DA was established based on the metabolomics data in two ion modes. For positive ions, the results showed that the BPH and control groups were well separated (Fig. 5a). The results are similar for negative ions (Additional file 2: Fig. S3a), which indicated that BPH significantly altered the rat intestinal metabolites. Differential metabolites were screened by multiple analysis and *t* test. The difference in screening results for positive and negative ions separately was shown by volcano maps (Fig. 5b, Additional file 2: Fig. S3b), resulting in 1167 differential metabolites, 563 of which were upregulated and 604 downregulated in the BPH group. Cluster analysis was performed for differential metabolites through Hierarchical Cluster and Euclidean Distance. The differential metabolite was shown as hierarchical cluster heatmaps (Fig. 5c, Additional file 2: Fig. S3c), indicating a difference between the two groups in intestinal metabolites.

#### BPH may cause changes in metabolic pathways

**Fig. 6:**
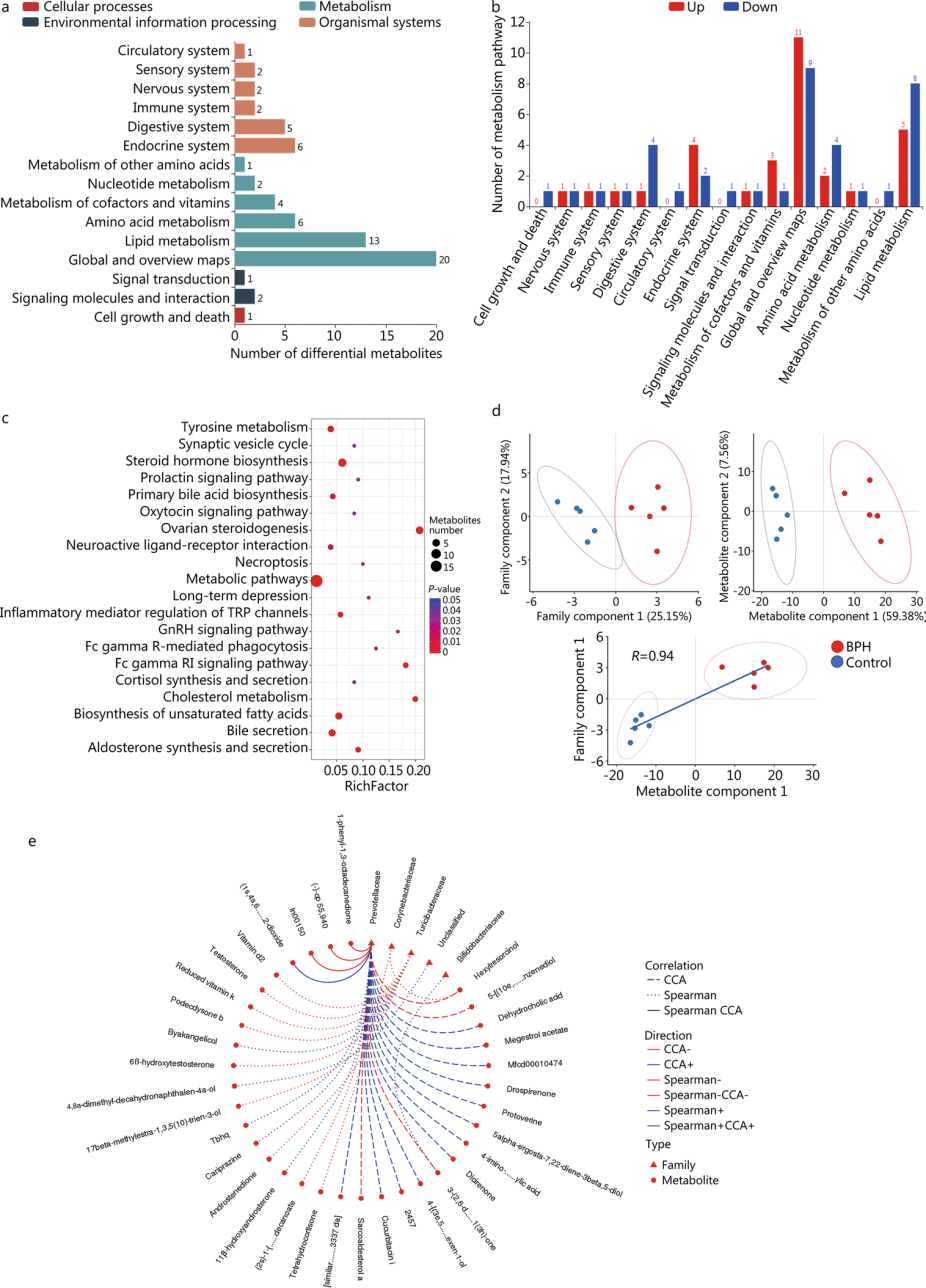
BPH leads to changes in metabolic pathways, which are related to changes in intestinal flora. **a** KEGG pathway function annotation bar graph of positive ion compounds: the X-axis represents the number of metabolite annotations, and the Y-axis represents the annotated KEGG pathway. **b** Statistical up-regulation and down-regulation of pathway classification of differential metabolites. **c** Bubble plots for metabolic pathway enrichment analysis: X-axis enrichment factor (RichFactor) is the number of differential metabolites annotated to the pathway divided by identified metabolites annotated to the pathway. The larger the value, the greater the proportion of differential metabolites annotated to the pathway. The dot size represents the number of differential metabolites annotated to this pathway. **d** Scatter plot of correlation between differential metabolites and microbial groups: (1) The component scatter plot of microbial group; (2) The component scatter diagram of differential metabolite; (3) Pearson correlation scatter diagram of the differential metabolite and the first component of microorganism group. The greater R is, the higher the degree of correlation between microorganism group and the first component of the metabolic pathway is. The color and ellipse represent sample groups. The greater the degree of sample dispersion in different groups, the better the classification effect of the component value. **e** Network diagram of correlation between differential metabolites and microbial groups at family level: the circle is the metabolite, the triangle is the microbial group; “ − ” represents negative correlation, “ + ” represents positive correlation. CCA: canonical correlation analysis, BPH: benign prostatic hyperplasia

To further understand the functions performed by these differential metabolites and their effects on the host. The identified differential metabolites were classified and annotated through KEGG database to clarify the functional characteristics and determine the main biochemical metabolic pathways and signal transduction pathways. For positive ions, the metabolic pathways and the number of metabolites corresponding to the differential metabolites are shown in Fig. 6a. The corresponding up-regulated and down-regulated metabolites are shown in the Fig. 6b. We found that the differential metabolites were mainly involved in cellular processes, environmental information processing, metabolism and organismal systems. For metabolism pathway, the most important pathways were global and overview maps (11 up-regulation and 9 down-regulation), lipid metabolism (5 up-regulation and 8 down-regulation), amino acid metabolism (2 up-regulation and 4 down-regulation), digestive system (1 up-regulation and 4 down-regulation) and endocrine system (4 up-regulation and 2 down-regulation). In addition, there were also circulator system, sensory system, nervous system and immune system pathways. The bubble diagram of metabolic pathway enrichment analysis (Fig. 6c) showed that the differential metabolites were significantly enriched in the following pathways: metabolic pathways, steroid hormone biosynthesis, ovarian steroidogenesis, biosynthesis of unsaturated fatty acids and bile secretion. Similar results were found for negative ion metabolites (Additional file 2: Fig. S4). The top 10 positive ion metabolic pathway enrichment ranking results are shown in the Additional file 1: Table S2.

#### Changes in metabolic pathways are associated with gut microbiota

To further explore the internal correlation between the metabolites differentially expressed in the samples and the microbial groups, we conducted correlation analysis at family levels.

Figure 6d not only shows the sample classification effect of differential metabolites and the first and second percentile scores of microbial groups, but also shows the correlation between the first component of differential metabolites and the first component of microorganisms. The Pearson correlation coefficient (*R* = 0.94) indicated a strong correlation between microbial groups and the first component of differential metabolites. The top 20 relationship pairs with the strongest rank correlation and the top 20 relationship pairs with the strongest canonical correlation were combined and presented by network graphs (Fig. 6e), which indicates *Prevotellaceae*, *Corynebacteriaceae*, *Turicibacteraceae*, *Bifidobacteriaceae* were significantly correlated with different metabolites.

At genus level, we found that different metabolites were significantly correlated with *Turicibacter*, *Prevotella*, *Coprococcus*, *Corynebacterium* and *Bilophila* (Additional file 2: Fig. S5a). At class level, we also found that different metabolites were significantly correlated with Coriobacteria, RF3, Betaproteobacteria, *Erysipelotrichi*, *Mollicutes*, and *Bacilli* (Additional file 2: Fig. S5b).

## Discussion

Because homeostasis of the microbiome is important for maintaining the health of the host, and the gut microbiota may change under disease conditions [29, 30], studying the response of microbial communities in the face of interference is critical. BPH has a high incidence rate in elderly men causing urination disorders, which seriously affect patients’ quality of life [10]. In addition to causing urinary dysfunction, in this study, we also found that BPH may have an impact on intestinal flora.

We found differences in the structure and function of the microbiota between the BPH and control groups. PCoA was used to compare the colonial differences. PCoA showed that the BPH group and the control group were clustered into two categories, indicating that BPH could affect the intestinal flora diversity of rats. By comparing the histogram of species classification, we found that there were significant differences in the flora between the two groups at the class family and genus levels, respectively. Compared with the control group, the level of *Firmicutes*, *Clostridiales*, *Clostridia*, *Coprococcus*, *Lachnospiraceae*, *Bacilli*, *Turicibacteraceae* are reduced and the level of *Corynebacteriaceae*, *Prevotellaceae*, *Prevotella*, *Corynebacterium*, *Bacteroidales* are increased in the BPH group.

The metabolic profile of the BPH group was significantly different from that of the control group. The sample points of the BPH and control groups were obviously separated in the PLS-DA, which indicates that BPH has an obvious effect on intestinal tract metabolism. Based on KEGG database, the metabolic pathways of differential metabolites were enriched and analyzed, and the most significant pathways were metabolic pathways, steroid hormone biosynthesis, and ovarian steroidogenesis. We concluded that there may be a gut-genitourinary axis to regulate the metabolic activities of the body.

More recently, the impact of metabolic factors for the development of BPH or LUTS has increasingly been recognized [31]. We found that *Firmicutes* decreased and *Bacteroidales* significantly increased in the BPH model. This is similar to a study showing that depression causes changes in gut microbiota [32], the study has found that *Firmicutes* was significantly reduced in the major depressive disorder groups compared with the healthy controls. Some studies suggest that BPH can cause symptoms of LUTS [33, 34], which has been shown to be associated with depression [35]. Another study has linked LUTS to the central nervous system [36]. It is suggested that BPH may affect changes of intestinal flora by causing abnormalities in the mental system. These findings enable a better understanding of changes in the gut microbiota composition in such patients, showing either a predominance of some potentially harmful bacterial groups or a reduction in beneficial bacterial genera. Further studies are warranted to elucidate the temporal and causal relationships between gut microbiota and depression and to evaluate the suitability of the microbiome as a biomarker.

Through experiments combined using the KEGG database, we found significant enhancement of steroid hormone biosynthesis and metabolic pathways in the intestinal tract (Additional file 1: Table S2). Some studies have shown a significant correlation between BPH and androgens [37, 38]. A previous study has shown that the metabolic diseases of diabetes can significantly reduce *Firmicutes* and *Clostridia* in the intestine, and *Betaproteobacteria* was highly associated with diabetic compared to non-diabetic persons [39]. This is similar to our findings that BPH could reduce intestinal *Firmicutes* and *Clostridia*, we also found that different metabolites had a significant correlation with *Betaproteobacteria*. These results indicate that BPH may change intestinal flora by affecting metabolic systems, such as glucose metabolism and hormone metabolism.

Studies have shown that the interaction of Omega-3 with gut microbes and immunity helps maintain intestinal wall integrity and interacts with the host immune system [40, 41]. Our study found that the metabolic pathways of biosynthesis of unsaturated fatty acids and inflammatory mediator regulation of transient receptor potential (TRP) channels were significantly altered in the BPH group, suggesting that BPH may affect the intestinal flora by affecting the immune system of the host or by mediating inflammation.

We found changes in the metabolic pathways that regulate bile secretion. Related studies have shown that bile acids are able to downregulate the expression of pro-inflammatory cytokines from monocytes, macrophages, dendritic cells and Kupffer cells [41, 42]. Moreover, free taurine generated by deconjugation of primary bile acids [43], can promote the activation of NOD-like receptor family pyrin domain containing 6 (NLRP6) inflammasome and the production of IL-18, supporting epithelial barrier function and maintenance [44]. This suggests that BPH may affect the immune system via the gastrointestinal system.

Our study found that, compared with the control group, the metabolic pathways related to tyrosine metabolism changed significantly in rat intestinal tracts in the BPH group. Tyrosine is the initial step in synthesis and the metabolic precursor of norepinephrine and epinephrine [45]. Overactivation of the ACE2-angiotensin 1–7/Mas receptor axis path increases the levels of the bioactive peptide hormone angiotensin II, which is associated with the development of BPH [46]. This evidence indicates that BPH might affect intestinal flora by regulating changes in intestinal metabolites, or even that intestinal flora may have an effect on the development of BPH by regulating changes in intestinal metabolites, thus forming a feedback pathway.

Recently, Takezawa et al*.* [47] included 128 patients who underwent prostate biopsies and reported that *Firmicutes*/*Bacteroidetes* ratio is associated with prostate enlargement. Also, we found that *Bacteroidetes* and *Firmicutes* were changed in BPH rats compared with the control group. This suggests that the occurrence and development of BPH may be strongly correlated with the alteration of *Bacteroidetes* and *Firmicutes*. Another study has reported that gut microbiota may be associated with ghrelin which plays an important role in activation of JAK2/STAT3 in BPH development, indicating ghrelin might be pathogenic factors for BPH and could be used as a target for mediation [48]. As we all know, ghrelin is a gastric hormone which could be affected by metabolic syndrome. Our previous study has proposed that metabolites, such as short‐chain fatty acids which produced by gut microbiota during fermentation of dietary fiber have similar chemical structures with hormone which is essential for the growth and survival of prostate cells [15]. That indicates metabolites may be correlated with prostate growth [15]. Ridlon et al. [49] has demonstrated that *Clostridium scindens*, a human gut microbe, has ability to convert glucocorticoids into androgens, which maybe promote development of BPH. In addition, the study found that microbe might contribute to the BPH-associated inflammation and tissue damage [17]. Cavarretta et al. [50] have also found that microbe could influence the tumor microenvironment of prostate cancer and promote tumor progression, another study suggests that bacterial metabolites (e.g. gingipains) may be involved in promoting the development of prostate cancer [51]. Other studies have found that microbe may affect the pathogenesis and progression of prostate cancer by regulating chronic inflammation, cytokines, apoptotic processes and hormonal production [52, 53]. This evidence suggests that the relationship of microbe and urinary diseases, such as BPH and prostate cancer, may be mediated by regulating the inflammation and microenvironment. In present study, compared to single-omics studies, the use of multi-omics methods of metabonomics provides a rich and complementary understanding of gut microbiota and helps researchers better understand the disease. Further research is needed to clarify the relationship between the two groups.

Testosterone was administered to create a BPH model rats for research [19, 20]. Several studies have explored the relationship between testosterone and gut microbiota. Harada et al. [54] have found that hypogonadism could alter cecal and fecal microbiota in cardiovascular disease male mice. Torres et al. [55] have reported that total testosterone may play a vital role in variation of gut microbiota in women with polycystic ovary syndrome, another study has found that increased *Parasutterella* abundance was positively correlated with serum testosterone level [56], while *Ruminococcaceae* was reported to be negatively correlated with testosterone level [57]. Conversely, the gut microbiota could also have an impact on testosterone level. It has been reported that *Escherichia*/*Shigella* strain could increase the concentration of testosterone in adult male mice [58]. Hydroxytyrosol could alter gut microbiota to benefit plasma metabolites, to enhance spermatogenesis and semen quality by raising testosterone and its derivatives [59]. Gut microbiota could also modulate the enterohepatic recirculation of testosterone, affecting the levels of sex steroid hormones and could generate androgens from glucocorticoids [60]. Based on previous studies, emerging evidence suggests the interplay between testosterone and gut microbiota extremely complicated, further studies are needed to explore the interaction between them.

From what has been discussed above, we found that BPH may affect intestinal flora and intestinal metabolites through a variety of pathways, including changing the proportion of intestinal flora through the nervous and the psychological systems. It affects intestinal flora and intestinal metabolites by regulating body metabolism and hormone synthesis and by mediating the inflammatory response of the host and the immune system to regulate the changes of intestinal flora. But our study also has some shortcomings. Our experimental sample size is small, and larger experimental samples are needed to verify and explain the association between BPH and microbiota in the future. In addition, BPH could cause complications such as prostatitis, renal impairment, and metabolic syndrome, these diseases have been found to be associated with the gut microbiota [61–63]. Therefore, future researches are needed to explore the relationship between them. Relevant clinical studies are also needed to further explore and verify these findings. At the same time, the detailed mechanism by which BPH affects intestinal flora and whether BPH causes pathophysiological changes in other systems still need to be further explored, which is what we're considering doing next.

## Conclusion

Our study shows that BPH is associated with alterations of abundance and diversity of gut microbiota and intestinal metabonomics in rats, which gives us a deeper understanding of disease. While the causal relationship between the gut microbiota and BPH is unclear. Thus, further studies are warranted to elucidate the potential mechanisms and causal relationships between BPH and gut microbiota.

## Supplementary Information

**Additional file 1: Table S1**. Comparison of 16S sequencing data volume between the two groups. **Table S2** Enrichment table of metabolism pathway.

**Additional file 2: Fig. S1**. 16S sequencing results of gut microbiota and gut microbiome composition at phylum level. **Fig. S2** Quality control of LC-MS/MS (**a-c**: positive ion, **d-f**: negative ion). **Fig. S3** BPH may induce variation in intestinal metabolites. **Fig. S4** BPH leads to changes in metabolic pathways. **Fig. S5** Association of intestinal differential metabolites with gut microbiota.

## Data Availability

The datasets generated and/or analysed during the current study are available in the Sequence Read Archive (SRA) repository, https://www.ncbi.nlm.nih.gov/, PRJNA762590.

## Abbreviations

AGC: Automatic gain control
arb: Arbitrary units
BPH: Benign prostatic hyperplasia
BPC: Base peak chromatogram
CCA: Canonical correlation analysis
COG: Clusters of orthologous genes
CV: Coefficient of variation
FLASH: Fast length adjustment of short reads program
HESI: Heated electrospray ionization
KEGG: Kyoto encyclopedia of genes and genomes
LC–MS/MS: Liquid chromatography tandem mass spectrometry
LDA: Linear discriminant analysis
LEfSe: LDA effect size
LUTS: Lower urinary tract symptoms
NLRP6: NOD-like receptor family pyrin domain containing 6
OTUs: Operational taxonomic units
PCA: Principal component analysis
PCoA: Principal coordinate analysis
PLS-DA: Partial least squares method-discriminant analysis
QC: Quality control
RDP: Ribosomal Database Project
SEM: Standard error of mean
TRP: Transient receptor potential
UPLC: Ultra performance liquid chromatography
UPGMA: Unweighted pair group method with arithmetic mean
VIP: Variable importance in projection
WGCNA: Weighted gene co-expression network analysis
