# Supplementary material for: Alterations of gut microbiota diversity, composition and metabonomics in testosterone-induced benign prostatic hyperplasia rats
Source: Mil Med Res. 2022 Mar 28;9:12. doi: 10.1186/s40779-022-00373-4 (PMC8962033; doi:10.1186/s40779-022-00373-4)
Supplement: Supplementary file 1 — Additional file 1: Table S1. Comparison of 16S sequencing data volume between the two groups. Table S2 Enrichment table of metabolism pathway. [file 40779_2022_373_MOESM1_ESM.doc]

**Table S1 Comparison of 16S sequencing data volume between the two groups**

| Reads | Control | | | | |  | BPH | | | | | | | |
| --- | --- | --- | --- | --- | --- | --- | --- | --- | --- | --- | --- | --- | --- | --- |
|  | Control 1 | Control 2 | Control 3 | Control 4 | Control 5 |  | BPH 1 | | BPH 2 | BPH 3 | | BPH 4 | BPH 5 | |
| Raw reads | 141,402 | 142,442 | 141,152 | 141,228 | 140,706 |  | 140,480 | 141,692 | | 141,902 | 141,488 | | | 142,030 |
| Clean reads | 136,892 | 137,272 | 135,802 | 136,046 | 136,148 |  | 136,268 | 136,794 | | 136,980 | 137,298 | | | 137,596 |
| Effective (%) | 96.81 | 96.37 | 96.21 | 96.33 | 96.76 |  | 97.00 | 96.54 | | 96.53 | 97.04 | | | 96.88 |
| OTUs | 389 | 386 | 477 | 462 | 442 |  | 483 | 429 | | 435 | 487 | | | 422 |

*OTUs* operational taxonomic units

**Table S2 Enrichment table of metabolism pathway**

| Pathway | Count | *P-*value | Pathway ID |
| --- | --- | --- | --- |
| Ovarian steroidogenesis | 5 | 1.23 **×** 10^-8^ | map04913 |
| Steroid hormone biosynthesis | 6 | 8.02 **×** 10^-7^ | map00140 |
| Metabolic pathways | 19 | 9.99 **×** 10^-7^ | map01100 |
| Biosynthesis of unsaturated fatty acids | 4 | 9.74 **×** 10^-5^ | map01040 |
| Bile secretion | 4 | 2.77 **×** 10^-4^ | map04976 |
| Cholesterol metabolism | 2 | 4.58 **×** 10^-4^ | map04979 |
| Fc epsilon RI signaling pathway | 2 | 5.59 **×** 10^-4^ | map04664 |
| Tyrosine metabolism | 3 | 2.08 **×** 10^-3^ | map00350 |
| Aldosterone synthesis and secretion | 2 | 2.30 **×** 10^-3^ | map04925 |
| Inflammatory mediator regulation of TRP channels | 2 | 5.75 **×** 10^-3^ | map04750 |

*TRP* transient receptor potential
