# Supplementary material for: Alterations of gut microbiota diversity, composition and metabonomics in testosterone-induced benign prostatic hyperplasia rats
Source: Mil Med Res. 2022 Mar 28;9:12. doi: 10.1186/s40779-022-00373-4 (PMC8962033; doi:10.1186/s40779-022-00373-4)
Supplement: Supplementary file 2 — Additional file 2: Fig. S1. 16S sequencing results of gut microbiota and gut microbiome composition at phylum level. Fig. S2 Quality control of LC-MS/MS (a-c: positive ion, d-f: negative ion). Fig. S3 BPH may induce variation in intestinal metabolites. Fig. S4 BPH leads to changes in metabolic pathways. Fig. S5 Association of intestinal differential metabolites with gut microbiota. [file 40779_2022_373_MOESM2_ESM.doc]

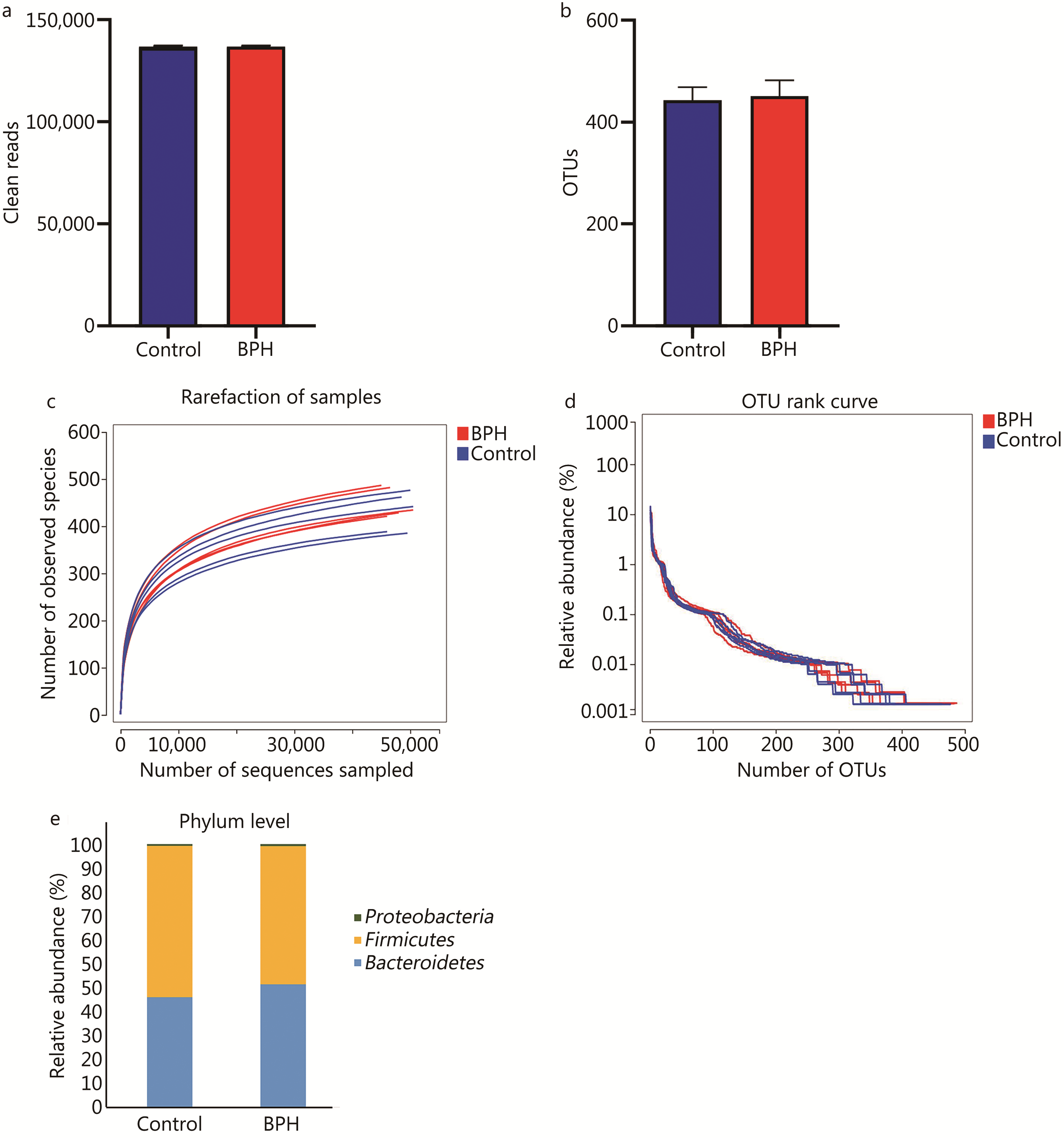


**Fig. S1** 16S sequencing results of gut microbiota and gut microbiome composition at phylum level. **a** Sequencing data of reads from the two groups. **b** OTUs obtained after sequencing data were clustered. Observed species curve (**c**) and OTU rank abundance curve (**d**) were used to assess the adequacy, evenness and richness of sequencing of each sample. Legends of different colors represent different groups. **e** Composition of fecal microbiome showing different communities at the phylum level. Results are expressed as mean ± SEM. BPH benign prostatic hyperplasia, OTUs operational taxonomic units


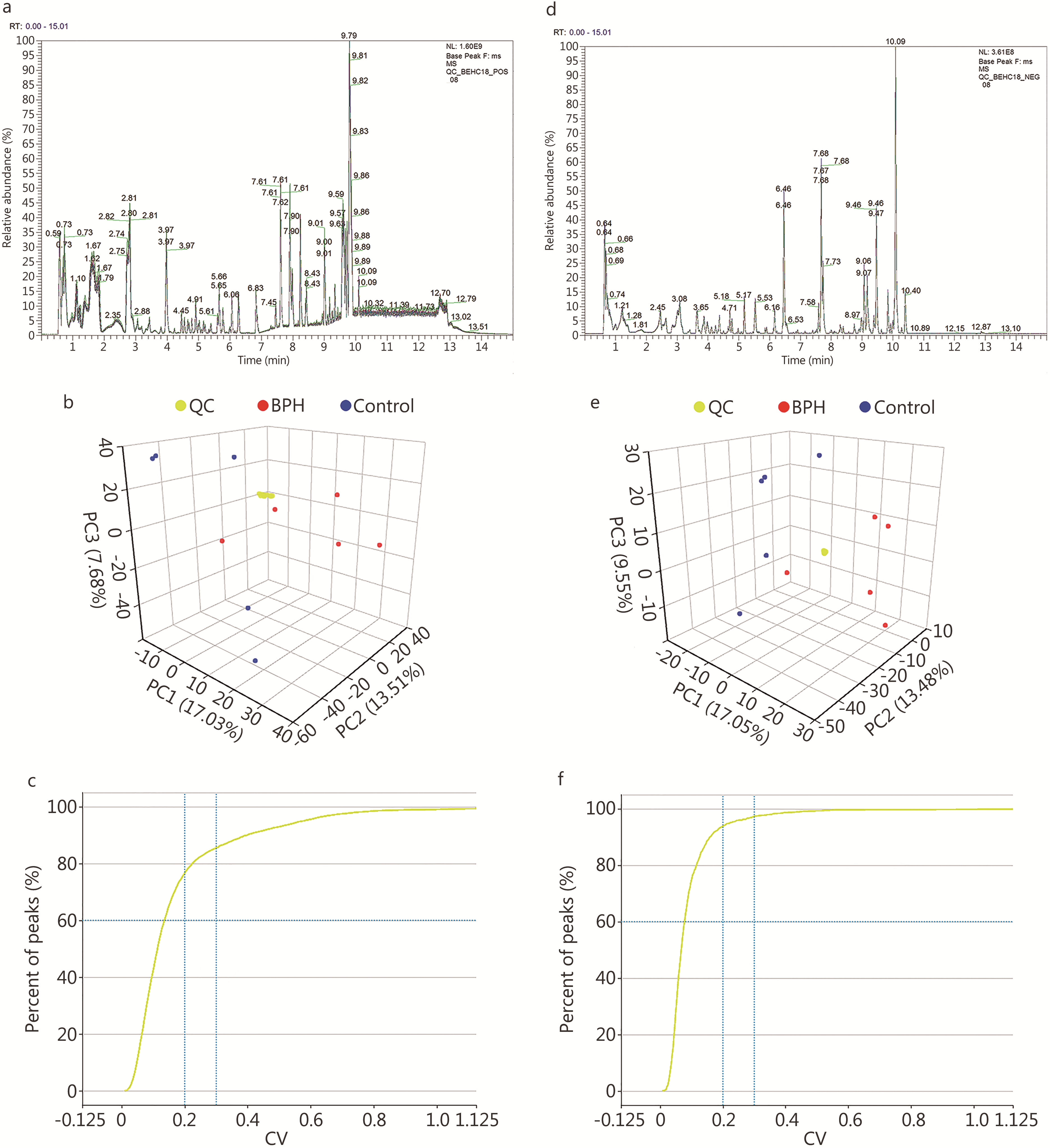


**Fig. S2** Quality control of LC-MS/MS (**a-c**: positive ion, **d-f**: negative ion). **a,** **d** QC sample BPC overlap diagram: a curve with time as the abscissa and the total ion intensity at each time point as the ordinate. The higher the degree of overlap, the more stable the instrument and the higher the data quality. **b,** **e** Principal component analysis (PCA) of QC sample: the better the QC aggregation of the quality control samples, the more stable the instrument and the better the repeatability of the collected data. **c,** **f** CV distribution of QC sample: in the QC group, the ions with CV less than 30% accounted for 60% as qualified. LC-MS/MS liquid chromatography tandem mass spectrometry, QC quality control, BPC base peak chromatogram, CV coefficient of variation


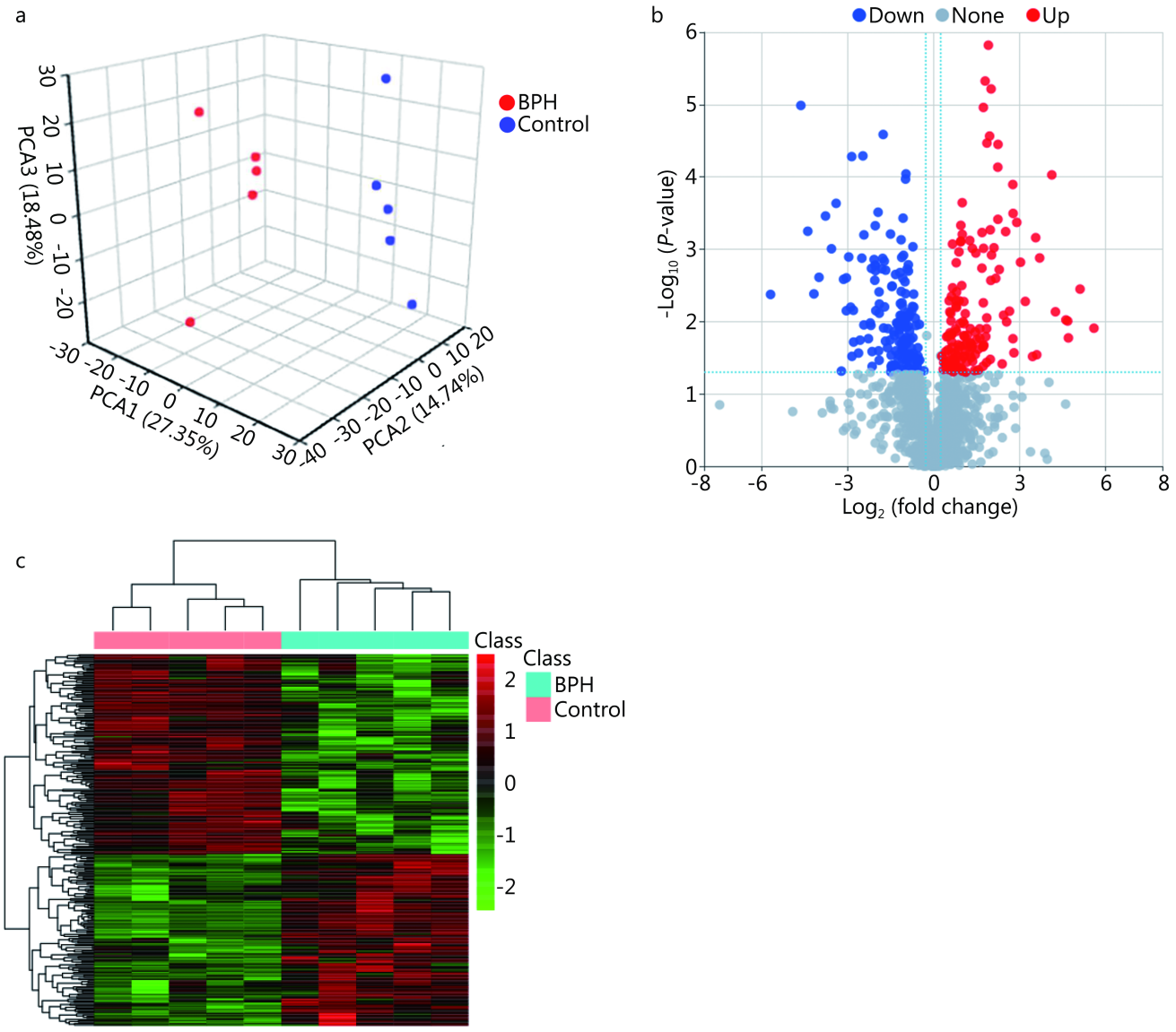


**Fig. S3** BPH may induce variation in intestinal metabolites. **a** PCA of negative ion. Each point represents a sample, and different groups are marked with different colors. **b** Volcano map of different metabolites (negative ion compound): blue represents the down-regulated differential metabolite, red represent the up-regulated differential metabolite, and metabolites without difference are labeled gray. **c** Cluster analysis of differential metabolites (negative ion compound): each row in the figure represents a differential metabolite, and each column represents a sample. The color represents the expression level, and the green to red corresponds to the expression level from low to high. BPH benign prostatic hyperplasia, PCA principal component analysis


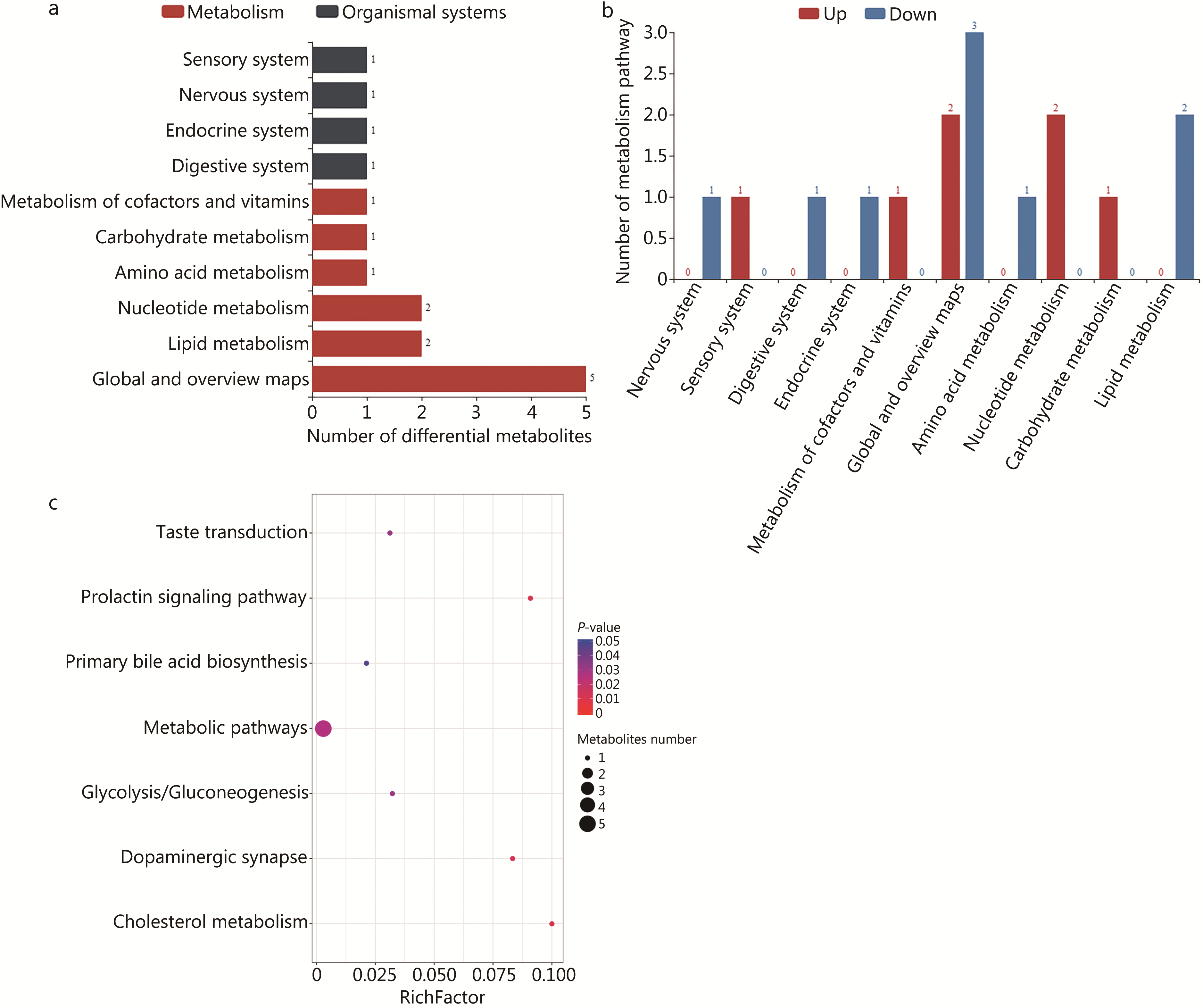


**Fig. S4** BPH leads to changes in metabolic pathways. **a** KEGG pathway function annotation bar graph of negative ion: the X-axis represents the number of metabolite annotations, and the Y-axis represents the annotated KEGG pathway. **b** Statistical up-regulation and down-regulation of pathway classification of differential metabolites. **c** Bubble plots for metabolic pathway enrichment analysis: X-axis enrichment factor (RichFactor) is the number of differential metabolites annotated to the pathway divided by identified metabolites annotated to the pathway. The larger the value, the greater the proportion of differential metabolites annotated to the pathway. The dot size represents the number of differential metabolites annotated to this pathway


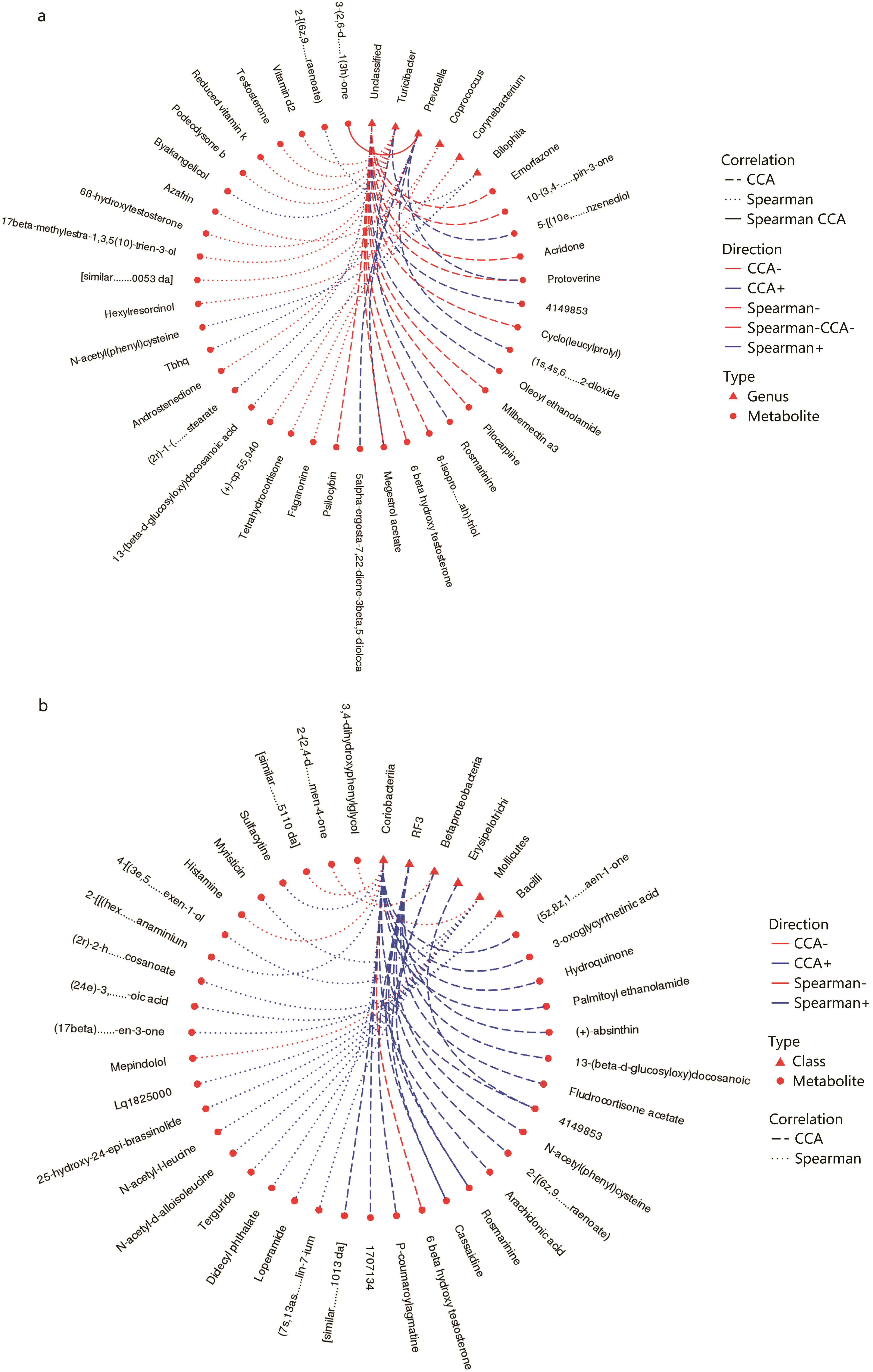


**Fig. S5** Association of intestinal differential metabolites with gut microbiota. **a** Network diagram of correlation between differential metabolites and microbial groups at genus level: the circle is the metabolite, the triangle is the microbial group. **b** Network diagram of correlation between differential metabolites and microbial groups at class level: the circle is the metabolite, the triangle is the microbial group; "-" represents negative correlation, "+" represents positive correlation. BPH benign prostatic hyperplasia
